# Specific features of l-histidine production by *Escherichia coli* concerned with feedback control of AICAR formation and inorganic phosphate/metal transport

**DOI:** 10.1186/s12934-018-0890-2

**Published:** 2018-03-15

**Authors:** Evgeniya A. Malykh, Ivan A. Butov, Anna B. Ravcheeva, Alexander A. Krylov, Sergey V. Mashko, Nataliya V. Stoynova

**Affiliations:** Ajinomoto-Genetika Research Institute, 1-st Dorozny pr., 1-1, Moscow, 117545 Russian Federation

**Keywords:** l**-**Histidine, Inorganic phosphate/metal transport, ATP-phosphoribosyltransferase, AICAR (ZMP), PitA, Pho regulon, *Escherichia coli*, l-Histidine production

## Abstract

**Background:**

In the l-histidine (His) biosynthetic pathway of *Escherichia* *coli*, the first key enzyme, ATP-phosphoribosyltransferase (ATP-PRT, HisG), is subject to different types of inhibition. Eliminating the feedback inhibition of HisG by the His end product is an important step that enables the oversynthesis of His in breeding strains. However, the previously reported feedback inhibition-resistant mutant enzyme from *E. coli*, HisG^E271K^, is inhibited by purine nucleotides, particularly ADP and AMP, via competitive inhibition with its ATP substrate. 5-Aminoimidazole-4-carboxamide ribonucleotide (AICAR), which is formed not only during His biosynthesis but also during de novo purine biosynthesis, acts as a natural analog of AMP and substitutes for it in some enzymatic reactions. We hypothesized that AICAR could control its own formation, particularly through the His biosynthetic pathway, by negatively influencing HisG enzymatic activity, which would make preventing ATP-PRT transferase inhibition by AICAR crucial for His overproduction.

**Results:**

For the first time, both the native *E. coli* HisG and the previously described feedback-resistant mutant HisG^E271K^ enzymes were shown to be sensitive to inhibition by AICAR, a structural analog of AMP. To circumvent the negative effect that AICAR has on His synthesis, we constructed the new His-producing strain EA83 and demonstrated its improved histidine production. This increased production was particularly associated with the improved conversion of AICAR to ATP due to *purH* and *purA* gene overexpression; additionally, the PitA-dependent phosphate/metal (Me^2+^-P_i_) transport system was modified by a *pitA* gene deletion. This His-producing strain unexpectedly exhibited decreased alkaline phosphatase activity at low P_i_ concentrations. AICAR was consequently hypothesized inhibit the two-component PhoBR system, which controls Pho regulon gene expression.

**Conclusions:**

Inhibition of a key enzyme in the His biosynthetic pathway, HisG, by AICAR, which is formed in this pathway, generates a serious bottleneck during His production. The constructed His-producing strain demonstrated the enhanced expression of genes that encode enzymes involved in the metabolism of AICAR to ATP, which is a substrate of HisG, and thus led to improved His accumulation.

**Electronic supplementary material:**

The online version of this article (10.1186/s12934-018-0890-2) contains supplementary material, which is available to authorized users.

## Background

l-Histidine (His) is an essential amino acid that is used in the pharmaceutical industry as a component of nutritious mixes for infant and adult humans [1]. In biochemical studies, His is a growth factor involved with many primary metabolites [2, 3]. Industrial His-producing strains of *Corynebacterium glutamicum*, *Brevibacterium flavum*, *Serratia marcescens* and *Escherichia coli* have been described [3].

The biosynthesis of His was initially well characterized in *Salmonella typhimurium* and *E. coli* as imperative model systems to study the fundamental processes connected with this function, including transcriptional attenuation, gene expression, and enzymatic feedback regulation [4–6].

**Fig. 1:**
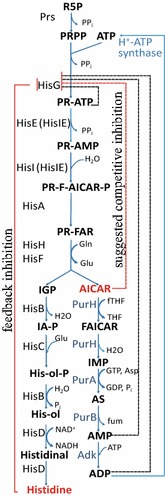
Metabolism of AICAR to ATP is an essential step in His synthesis. *Prs* ribose-phosphate diphosphokinase, *ATP* adenosine triphosphate, *R5F* ribose 5-phosphate, *PRPP* phosphoribosyl pyrophosphate, *PR-ATP* phosphoribosyl-ATP, *PR-AMP* phosphoribosyl-AMP, *PR-F-AICAR-P* phosphoribosylformimino-AICAR-phosphate, *PR-FAR* phosphoribulosylformimino-AICAR-phosphate, *AICAR* 5-aminoimidazole-4-carboxamide ribonucleotide, *FAICAR* 5′-phosphoribosyl-5-formamido-4-imidazole carboxamide, *IMP* inosine 5′-phosphate, *AS* adenylosuccinate, *AMP* adenosine monophosphate, *ADP* adenosine diphosphate, *IGP* imidazole glycerol phosphate, *IA-P* imidazole acetol-phosphate, *His-ol-P* l-histidinol-phosphate, *His-ol* histidinol, *Histidine* l-histidine, *HisG* ATP-phosphoribosyltransferase, *HisIE* bifunctional enzyme (*HisE* phosphoribosyl-ATP pyrophosphatase, *HisI* phosphoribosyl-AMP cyclohydrolase), *HisA* phosphoribosylformimino-5-amino-1-phosphoribosyl-4-imidazole carboxamide isomerase, *HisHF* IGP synthase, *HisB* bifunctional imidazoleglycerol-phosphate dehydratase/histidinol-phosphatase, *HisC* histidinol-phosphate aminotransferase, *HisD* bifunctional histidinal dehydrogenase/histidinol dehydrogenase, *PurH* bifunctional AICAR transformylase/IMP cyclohydrolase, *PurA* adenylosuccinate synthetase, PurB adenylosuccinate lyase, *Adk* adenylate kinase, *Gln* glutamine, *Glu* glutamate, *fTHF* formyltetrahydrofolate, *THF* tetrahydrofolate, *Asp* aspartate, *GTP* guanosine-triphosphate, GDP guanosine-diphosphate, *PP*_*i*_ inorganic pyrophosphate, *P*_*i*_ inorganic phosphate, *NAD*^*+*^*/NADH* nicotinamide adenine dinucleotide oxidized/reduced form, *fum* fumaric acid. Feedback inhibition by His is indicated by red line; competitive inhibitions by ATP-PRT, AMP and ADP are indicated by dot lines; suggested competitive inhibition by AICAR is indicated by the red dot line

In bacterial cells, the His biosynthesis pathway is associated with ten biochemical reactions and nine enzymes that contribute to the conversion of two biosynthetic precursors, phosphoribosyl pyrophosphate (PRPP) and adenosine triphosphate (ATP), into this amino acid (Fig. 1) [6].

The first key step in the His biosynthetic pathway, the condensation of PRPP and ATP to form phosphoribosyl-ATP (PR-ATP), is catalyzed by the ATP-PR transferase (HisG, EC 2.4.2.17), which is exposed to a variety of types of inhibition: allosteric feedback inhibition by His, competitive inhibition by adenosine mono- and diphosphates (AMP and ADP, respectively) [7], and competitive product inhibition by PR-ATP. Eliminating the His-mediated feedback inhibition of HisG plays a critical role in the regulation of His biosynthesis [6, 8]. Feedback-resistant HisG-mutant enzymes from *S. typhimurium* [9] and *C. glutamicum* [10, 11] have been previously characterized. The HisG^E271K^, Fbr-mutant from *E. coli* was obtained by traditional selection methods several decades ago [12], and its properties were later investigated [13]. Despite having complete resistance to feedback inhibition by His, this mutant enzyme is still susceptible to competitive inhibition by purine nucleotides, particularly ADP and AMP [12]. This finding suggests that HisG^E271K^, similar to the native HisG, could be the target of inhibition by the AMP structural analog, 5-aminoimidazole-4-carboxamide-1-beta-d-ribofuranosyl 5′-monophosphate (AICAR). Figure 1 indicates that AICAR is formed via the His biosynthetic pathway as well as through the de novo purine biosynthetic pathway [6, 14]. In the present study, we confirmed that both the native (HisG^WT^) and feedback-resistant (HisG^E271K^) enzymes were sensitive to inhibition by AICAR. The formation of AICAR through the His biosynthetic pathway in *E. coli* is thus negatively controlled by end-product (AICAR) inhibition of the first key enzyme in the pathway, HisG. Considering that His biosynthesis is accompanied by equimolar AICAR generation, the effective metabolism of AICAR to ATP, which in turn functions as the HisG substrate, appears crucial for the production of this amino acid.

The reactions that convert AICAR into ATP include the use of bi-functional PurH (EC 2.1.2.3 + EC 3.5.4.10) [15], PurA (EC 6.3.4.4) [16], PurB (EC 4.3.2.2.), Adk (EC 2.7.4.3.) and H^+^-ATP synthase (F_1_F_0_-ATP synthase) [17] (Fig. 1). Since the final step of ATP synthesis from AICAR includes ADP phosphorylation by inorganic phosphate (P_i_) that is catalyzed by the proton force-dependent ATP synthase, the maintenance of intracellular P_i_ at the appropriate level is a significant requirement for the efficiency of the whole pathway.

The two major P_i_ transport systems were initially described in *E. coli* cells: Pst (PstSCAB, phosphate-specific transport) and Pit (PitA, phosphate inorganic transport) [18–21]. PstSCAB, a member of the ABC superfamily of ATP-dependent transporters, is the major high-affinity, low-velocity phosphate uptake system. Under conditions of P_i_ limitation, the Pst system is activated more than 100-fold, and P_i_ is primarily taken up by the Pst transporter. In addition, at basal expression levels, the Pst system and not PitA (see below) primarily contributes to P_i_ uptake when excess P_i_ is present [22]. *E. coli* also possesses a high-velocity, low-affinity P_i_ transporter, PitA, which does not belong to the phosphate (Pho) regulon; PitA is dependent on the proton motive force for energization, and P_*i*_ uptake via PitA is defined by the presence of divalent cations such as Zn^2+^, Mg^2+^, Ca^2+^, Co^2+^, and Mn^2+^, which form soluble metal phosphate (MeHPO_4_) complexes [23]. PitA gene expression was reported to be modulated in a chemically defined medium by the availability of P_i_ and Zn^2+^ ions [24], and PitA functions primarily as a transporter of divalent metal cations (Zn^2+^) complexed with P_i_ [22, 25]. A third P_i_ transport gene, *pitB*, encodes a functional P_i_ transporter that is a homolog of PitA, sharing 81% identity (http://blast.ncbi.nlm.nih.gov/, BLAST^®^ data); *pitB* is repressed at low P_i_ levels by the Pho regulon [26] and likely does not play a role in P_i_ uptake in normal cells because it is not expressed under normal growth conditions [22]. Two additional transporters, encoded by *glpT* and *uphT*, transport P_i_ with low affinity [27–29]; however, in the absence of Pst and Pit, these systems cannot support growth when phosphate is provided as P_i_ [24, 30]. Maintaining a sufficient P_i_ pool is extremely important for many energy-consuming cellular processes [18], particularly for His synthesis.

Oversynthesis of any cell metabolite, e.g., His, alters the carbon fluxes and pools of intermediate compounds, possibly resulting in consequences that are generally unpredictable. In the present study, some effects were revealed to be related to His overproduction by *E. coli*, and the elucidation of these effects has begun. AICAR negatively influenced His biosynthesis in *E. coli*, and a strategy for the efficient conversion of this intermediate to ATP by enhancing the de novo purine biosynthetic pathway genes, *purH* and *purA,* was developed that improved His production. The influence of AICAR on the activity of HisG was studied and revealed that AICAR is a structural analog of AMP; AICAR was even a stronger inhibitor of HisG (K_i_ = 0.65 mM) than AMP (K_i_ = 2.15 mM). His oversynthesis was accompanied with a change in the functioning of the Pho regulon that was explained by changes in the AICAR pool. Furthermore, *pitA* gene deletion positively influenced His overproduction, and although the precise mechanism underlying this effect remains unclear, several explanations based on biological role of PitA as a Me^2+^-P_i_ importer were supposed.

## Methods

### Strains, plasmids and media

All bacterial strains and plasmids used in this study are listed in Table 1.

**Table 1:** *E. coli* strains and plasmids used in this study

Strain or plasmid	Description	Source
Strains		
MG1655	*Escherichia coli* K-12 wild-type	VKPM^a^ B-6195
BL21(DE3)	*E. coli* B F^−^ *ompT gal dcm lon hsdS*_*B*_ (*r*_*B*_^−^*m*_*B*_^−^) λ(DE3 [*lacI* P_*lac*UV5_-*T7gene1 ind1 sam7 nin5*]) [*malB*^+^]_K–12_ (λ^S^)	[32]
KF37	MG1655^+^-[Δ*purR* P_*his*_-Δ*hisL*′ *hisG*^E271K^*DCBHAFI*]	[13]
MG1655 [Δ*purR*::Cm^R^]	MG1655 wild-type *E. coli* K-12, but Δ*purR*::λ*attR*-*cat*-λ*attL*	Laboratory collection
BW25113 [Δ*purH*::Km^R^]	*lacI*^q^ *rrnB*_T14_ Δ*lacZ*_WJ16_ *hsdR514* Δ*araBAD*_AH33_ Δ*rhaBAD*_LD78_, Δ*purH*::*FRT*-*kan*-*FRT*	[33]
MG1655 [Δ(φ80-*attB*)]	MG1655 with deleted native (φ80-*attB*) site	[34]
MG1655 [Δ(φ80-*attB*) IS 5.11::φ80-*attB*]	MG1655 with deleted native φ80 *attB* site and reconstruction of *attB* site in IS 5.11 locus	[34]
MG1655 [Δ(φ80-*attB*) IS 5.11::Cm^R^-P_*tac*21_-*purA*]	MG1655-[Δ(φ80-*attB*) IS 5.11::φ80-*attB*], but IS 5.11::(λ-*attB*) P_*tac*21_-*purA*	This study
MG1655 [Cm^R^-P_L_-*purH*]	MG1655, but λ*attR*-*cat*-λ*attL*-P_L_-*purH*	This study
MG1655 [Δ*pitA*::Km^R^]	MG1655, but Δ*pitA*::λ*attR*-*kan*-λ*attL*	This study
MG1655 [Cm^R^-P_L*tac*_-*pitA*]	MG1655, but λ*attR*-*cat*-λ*attL*-P_L*tac*_-*pitA*	This study
MG1655 [Δ*purR*::Cm^R^ Δ*purH*:: Km^R^]	MG1655, but Δ*purR*::λ*attR*-*cat*-λ*attL* and Δ*purR*::FRT-*kan*-FRT	This study
KF37 [Δ*pitA*::Km^R^]	KF37, but Δ*pitA*::λ*attR*-*kan*-λ*attL*	This study
KF37 [IS5.11::Cm^R^-P_*tac*21_-*purA pitA*^−^	KF37, but IS 5.11:: λ*attR*-*cat*-λ*attL* P_*tac*21_-*purA pitA*^−^	This study
KF37 [IS5.11::Cm^R^-P_*tac*21_-*purA pitA*^+^]	KF37, but IS 5.11::λ*attR*-*cat*-λ*attL* P_*tac*21_-*purA pitA*^+^	This study
EA79	KF37, but IS 5.11::(λ-*attB*) P_*tac*21_-*purA pitA*^−^	This study
EA83	EA79, but (λ-*attB*) P_L_-*purH*	This study
CC118 λ*pir*+	Host strain for maintenance of *pir*-dependent recombinant plasmids	[35]
MG1655 [Δ(φ80-*attB*) ∆*yibH*::φ80-*attB*]	MG1655 with deleted native φ80 *attB* site and reconstruction of *attB* site instead of *yibH* locus	This study
MG1655 [∆*yibH*::Tc^R^-*phoB*^DBD^]	MG1655 [Δ(φ80-*attB*) ∆*yibH*::φ80-*attB*], but ∆*yibH*::λ*attR*- *tetAR*-λ*attL*-*phoB*^DBD^	This study
MG1655 [∆*yibH*::Km^R^-P_*lac*UV5_-*phoB*^DBD^]	MG1655 [Δ(φ80-*attB*) ∆*yibH*:: λ*attR*-*tetAR*-λ*attL* -*phoB*^DBD^], but ∆*yibH*::λ*attR*-*kan*-λ*attL*-P_*lac*UV5_-*phoB*^DBD^	This study
KF37 [∆*yibH*::Tc^R^-*phoB*^DBD^]	KF37, but ∆*yibH*::λ*attR*- *tetAR*-λ*attL*-*phoB*^DBD^	This study
KF37 [∆*yibH*::Km^R^-P_*lac*UV5_-*phoB*^DBD^]	KF37, but ∆*yibH*::λ*attR*-*kan*-λ*attL*-P_*lac*UV5_-*phoB*^DBD^	This study
Plasmids		
pKD46	oriR101, repA101ts, *araC*, P_*araB*_-[γ, β, exo of phage λ], Ap^R^; used as a donor of λRed-genes to provide λRed-dependent recombination	[36]
pMWts-λInt/Xis	oriR101, repA101ts, λcIts857, λP_R_→λ*xis*-*int*, Ap^R^; used as a helper plasmid for thermoinducible expression of the λ *xis*-*int* genes	[34]
pAH123	oriR101, repA101ts, λcIts857, λP_R_→φ80-*int*, Ap^R^; used as a helper plasmid for thermoinducible expression of the φ80-*int* gene	[37]; GenBank accession number AY048726
pET15b	Ap^R^, pBR322 origin, P_*lacI*_-*lacI*, and T7 promoter/O_*lac*_, T7 transcription start, His6-Tag coding sequence, T7 terminator	Novagen
pMW118-Km^R^	oriR101, repA, MCS, Ap^R^, λ*attR*-*kan*-λ*attL*—donor of λXis/Int-excisable Km^R^ marker	[38]
pET15b-*hisG*	Ap^R^, pET15b containing *hisG* gene	This study
pET15b-*hisG*^E271K^	Ap^R^, pET15b containing the mutant *hisG*^E271K^ gene	This study
pAH162-Tc^R^-2Ter	attP *phi80,* pAH162, λ*attL*-*tetA*-*tetR*-λ*attR*	[34]; Gene Bank accession number AY048738
pMW119-P_*lac*_-*lacI*	Ap^R^, low-copy-number vector pMW119, containing P_*lac*_-*lacI*	Laboratory collection
pMW119-P_*lac*_-*lacI*-*purA*	Ap^R^, low-copy-number vector pMW119, containing P_*lac*_-*lacI*-*purA*	This study
pAH162-Tc^R^-2Ter-*purA*	*oriR*γ, φ80-*attP*, λ*attL*-*tetA*-*tetR*-λ*attR*, *purA*	This study
pML-P_*lac*UV5_-*lacI*	*P*_*lac*UV5_-*lacI*, rep(pMB1), Ap^R^, Cm^R^	[39]
pAH162-Tc^R^-2Ter-*phoB*^DBD^	*oriR*γ, φ80-*attP*, λ*attL*-*tetA*-*tetR*-λ*attR*, promoter-less *phoB*^DBD^	This study

*Ap*^*R*^ ampicillin resistance, *Cm*^*R*^ chloramphenicol resistance, *Tc*^*R*^ tetracycline resistance

^a^VKPM Russian National Collection of Industrial Microorganisms

The following media were used to culture bacteria: Luria–Bertani (LB), M9, SOB, SOC [31], and MOPS, and the MOPS medium was supplemented with 0.25 mM KH_2_PO_4_. Glucose (0.4%) was added to minimal media as a carbon source. The antibiotics ampicillin (Ap, 100 mg/L), chloramphenicol (Cm, 20 mg/L), tetracycline (Tc, 20 mg/L), and kanamycin (Km, 50 mg/L) were used when necessary.

### Test tube cultivation conditions

For His accumulation, the strains were grown in LB medium at 30 °C overnight; 0.1 mL of each culture was then inoculated into 2 mL of fermentation medium in a test tube, which was then cultivated for 72 h at 30 °C with shaking on a rotary shaker (250 rpm), until all of the glucose was consumed. The composition of the fermentation medium (g/L) was as follows: soybean meal hydrolysate, 0.1 g; l-aspartate, 0.5 g; (NH_4_)_2_SO_4_, 9 g; KCl, 0.5 g; KH_2_PO_4_, 0.25 g; MgSO_4_·7H_2_O, 0.2 g; FeSO_4_·7H_2_O, 0.01 g; MnSO_4_·5H_2_O, 0.01 g; ZnSO_4_·7H_2_O, 0.01 g; adenosine, 0.1 g; vitamin B1, 0.0005 g; betaine, 1 g; CaCO_3_, 30 g; and glucose, 25 g, as the carbon source; the pH was adjusted to 6.0.

### DNA manipulation

Genetic manipulation of *E. coli* and techniques for the isolation and manipulation of nucleic acids were performed according to standard protocols [31]. Restriction enzymes, T4 DNA ligase, High Fidelity PCR Enzyme Mix, Taq polymerase, and 1-kb DNA Ladder were purchased from Thermo Scientific Inc. (USA). Plasmids and genomic DNA were isolated using QIAGEN Plasmid Mini Kits (QIAGEN GmbH, Germany) and Bacterial Genomic DNA Kits (Sigma), respectively. QIAquick Gel Extraction kits (QIAGEN GmbH, Germany) were used to isolate DNA from agarose gels. Oligonucleotides were purchased from Evrogen (Russia). The sequences of the oligonucleotide primers are presented in Additional file 1: Table S1.

### Quantitative determination of the l-histidine concentration

The amount of His that accumulated in the medium was determined by thin-layer chromatography (TLC) using plates coated with silica gel (Merck, Germany). Samples were applied to the TLC plates using Linomat 5 (Camag, Switzerland). The plates were developed with a mobile phase consisting of propan-2-ol:acetone:25% aqueous ammonia:water = 12.5:12.5:3:2 (v/v). A solution of ninhydrin (1%) in acetone was used as the visualizing reagent; the plates were dried and then scanned at 520 nm using a Linomat 5 scanner (Camag, Switzerland).

### pET15b-*hisG* plasmid construction

The native *hisG* gene was cloned into a pET15b plasmid vector after PCR amplification (see Additional file 1: Table S1 for details, primers 4, 5). The obtained PCR fragments were treated with *Bam*HI and *Nde*I endonucleases and ligated into a pET15b vector that had been treated with the same enzymes. The obtained pET15b-*hisG* plasmid carries the gene encoding HisG with an N-terminal cleavable His_6_-Tag (HT-HisG) for affinity purification. The obtained plasmid was introduced into the strain *E. coli* BL21(DE3) for HT-HisG induced biosynthesis followed by tagged-protein purification. Plasmid pET15b-*hisG*^E271K^, which harbors the mutant feedback-resistant His_6_-tagged HisG (HT-HisG^E271K^) gene, was obtained in a similar manner.

### HisG expression and purification

*Escherichia coli* BL21(DE3)/pET15-*hisG* was grown in 50 mL of LB medium until the OD_560_ was 0.8. Protein expression was induced by the addition of isopropyl β-d-1-thiogalactopyranoside (IPTG) to a final concentration of 1 mM, which was followed by incubation for 4 h. The cells were centrifuged, washed twice with 50 mL of 100 mM NaCl solution, centrifuged again, resuspended in 50 mL of buffer I (300 mM Tris–HCl, 300 mM KCl, and 1 mM PMSF, pH 8.1) and disrupted by two passages through a cold French press cell at 2000 psi. Unbroken cells and cell debris were removed by centrifugation at 12,000 rpm for 30 min at 4 °C. The supernatant was applied to a 1-mL HiTrap^®^ column (Pharmacia, Sweden); this column was then washed with 10 ml of buffer I, and bound protein was eluted with a linear gradient of buffer I and buffer II (20 mM Tris–HCl and 400 mM imidazole, pH 8). The resulting HT-HisG preparation was further purified by gel filtration using a 10-mL BioGel P10 column (Pharmacia, Sweden) equilibrated with buffer III (20 mM potassium phosphate buffer, pH 7, 1 mM DTT, 10 μM PLP, and 10% (w/v) glycerol). This method was used to purify two ATP-phosphoribosyltransferases (ATP-PRTs): native HT-HisG and mutant HT-HisG^E271K^. The purity of the obtained proteins was greater than 90% according to SDS-PAGE analysis (see Additional file 2: Figure S1).

### ATP-phosphoribosyltransferase assay

The HisG enzymatic activity and initial velocity of the forward phosphoribosyltransferase reaction were measured by monitoring the formation of PR-ATP (ε_290_ = 3600/M/cm [40]) in the presence of *E. coli* inorganic pyrophosphatase (PPase, Sigma-Aldrich, USA). Reactions were performed in UV-star 96-well microplates (Greiner Bio-One, Germany). The reaction mixture consisted of 100 mM Tris–HCl (pH 8.1), 100 mM KCl, 10 mM MgCl_2_, 5 mM ATP, 1 mM PRPP, 10 mU of pyrophosphatase and 500 nM *E. coli* HisG [41]. The reaction was initiated by the addition of ATP. The absorption at 290 nm was monitored for 30 min in 2-min intervals (Tecan, Switzerland). A reaction mixture containing water instead of ATP substrate was used as a blank control. To test the inhibition of HisG by AMP and AICAR, these compounds were added to the initial reaction mix. Notably, the molar extinction coefficients of AICAR and AMP are ε_290, AICAR_ = 2700/M/cm and ε_290, AMP_ = 240/M/cm. AICAR concentrations higher than 1 mM were therefore undesirable for the measurements, but AMP concentrations as high as 5 mM could be used without significant interference. To obtain the values of K_I, AMP_ and K_I, AICAR_, AMP or AICAR was added to the reaction mix to a final concentration of 20 and 1 mM, respectively. To calculate the inhibition constants, the following formula was used [42]: *K*_*I*_ = [*I*]/((*K*_*Mi*_/*K*_*M*_) − 1), where *K*_*I*_ is the inhibition constant, [*I*] is the concentration of the inhibitor, *K*_*M*_ is the Michaelis constant, and *K*_*Mi*_ is the *K*_*M*_ value in the presence of inhibitor.

### Alkaline phosphatase (PhoA) assay

The enzymatic activity of *E. coli* alkaline phosphatase (the *phoA* gene protein product, E.C. 3.1.3.1) was measured according to the method of Brickman and Beckwith [43] with modifications. Cells were grown in a flask for 24 h in MOPS medium supplemented with 0.4% glucose and 0.25 mM KH_2_PO_4_ and were then washed with 0.9% NaCl. The cells were then lysed by sonication, and cell debris was removed by centrifugation at 12,000 rpm for 20 min at 4 °C. The protein concentration in the supernatant was determined using a standard Bradford Protein assay [44]. PhoA enzymatic activity was analyzed 2 or 24 h after P_i_ exhaustion. Enzyme reactions were performed in 96-well UV-star microplates (Greiner Bio-On, Germany) and consisted of 500 mM Tris–HCl, pH 8.0, 1 mM MgCl_2_, and supernatant (or supernatant diluted in 0.9% NaCl) containing from 0.02 to 0.10 mg of protein. *p*-Nitrophenyl-phosphate (pNPP) was used as the substrate. After pNPP addition, the reaction mixture was incubated at 37 °C for 3–4 min, by which time it had turned yellow. The reaction was stopped by the addition of 1 M KH_2_PO_4_, and the absorbance at 410 nm was measured in a UV-star microplate cell against a control reaction that did not contain protein.

### Inorganic P_i_ measurement

The amount of P_i_ in culture media was determined using a common method based on phosphomolybdate reduction to molybdenum blue [45, 46]. In 96-well microplates containing 0.1 mL of each sample to be analyzed, 0.075 mL of ammonium molybdate-ferrous sulfate (a colored mixture) was added, and the resulting solution was vortexed and incubated at room temperature for 10 min. The colored mixture was prepared daily by mixing 4 vol of 2.5% ammonium molybdate solution with 1 vol of 2.5% ferrous sulfate solution. For the calibration curve, the colored mixture was added to samples containing standard solutions of KH_2_PO_4_. The absorbance was measured at 700 nm. A sample without added P_i_ served as a blank.

### Construction of strains and plasmids

Insertions and deletions in the chromosome of *E. coli*, usually the MG1655K-12 strain, were prepared via λ-Red modification according to the method of Datsenko and Wanner [36], exploiting the λXis/Int system for marker excision [34]. The plasmid pKD46, carrying the arabinose-inducible λ-Red genes [36] and kindly gifted by Dr. Wanner, was used. $\upvarphi$80-mediated site-specific integration was carried out according to the method of Haldimann and Wanner with the thermoinducible $\upvarphi$80-Int gene in the pAH123 plasmid [37]. Specially designed cassettes with Cm^R^, Km^R^ and Tc^R^ markers bracketed by hybrid λ*attP*/λ*attB*-sites (λ*attL* and λ*attR*) were transferred into *E. coli* strains by P1-mediated general transduction (P1-duction) [47]. Finally, Cm^R^, Km^R^ and Tc^R^ were eliminated from the *E. coli* chromosome using a λXis/Int site-specific recombination system with pMWts-λInt/Xis as a helper plasmid [34].

#### Construction of *E. coli* strain MG1655 [Δ($\upvarphi$80-*attB*) IS 5.11::Cm^R^-P_*tac*21_-*purA*]

To construct *E. coli* strain MG1655 [Δ($\upvarphi$80-*attB*) IS5.11::Cm^R^-P_*tac*21_-*purA*], the native *purA* gene was PCR amplified with primers 6–7 (see Additional file 1: Table S1 for details) using the MG1655 DNA as a template and was then cloned into a pMW119-P_*lac*_-*lacI* vector that had been treated with *Sma*I. Plasmid pMW119-P_*lac*_-*lacI*-*purA* was treated with *Bam*HI and *Kpn*I endonucleases, and the resulting *purA*-containing fragment was recloned into a pAH162-Tc^R^-2Ter [34] integrative plasmid. The resulting plasmid, pAH162-Tc^R^-2Ter-*purA*, was used for $\upvarphi$80-mediated integration of the promoter-less *purA* gene into the artificial $\upvarphi$80-*attB* site of the MG1655 [Δ($\upvarphi$80-*attB*) IS 5.11::$\upvarphi$80-*attB*] strain constructed earlier [34]. Expression of the *purA* gene was activated by λRed-mediated insertion of the P_*tac*21_ promoter region with two point mutations in the -35 region (*ttgaca* of P_*tac*_ was replaced with *tttgca*) upstream of the gene using primers 8–9 (see Additional file 1: Table S1 for details). The obtained strain MG1655 [Δ($\upvarphi$80-*attB*) IS5.11::Cm^R^-P_*tac*21_-*purA*] was used as the donor of a *purA*-carrier expression cassette, marked by an excisable Cm^R^-marker, for P1-duction of the corresponding gene in the different strains of interest.

#### Construction of *E. coli* strain MG1655 [Cm^R^-P_L_-*purH*]

To construct *E. coli* strain MG1655 [Cm^R^-P_L_-*purH*], the upstream region of the *purH* gene, which is associated with *purD* in a single operon, was modified by replacement of the native regulatory region with the λP_L_ promoter by λRed recombination as mentioned above. To construct the PCR fragment for λRed recombination with an excisable *cat* marker and flanking sequences homologous to the *purHD* regulatory region, we used primers 10 and 11 (see Additional file 1: Table S1 for details).

#### Construction of strain MG1655 [*∆pitA*::Km^R^]

To delete *pitA* in *E. coli* strain MG1655, the λRed integration method was used as described above. To construct the PCR fragment for λRed recombination with an excisable *kan* marker and flanking sequences homologous to the *pitA* gene, we used primers 12 and 13 (see Additional file 1: Table S1 for details) with the pMW118-Km^R^ plasmid as a template. The chromosomal deletion of *pitA* was confirmed by PCR.

#### Construction of *E. coli* strain MG1655 [Cm^R^-P_L*tac*_-*pitA*]

To construct *E. coli* strain MG1655 [Cm^R^-P_L*tac*_-*pitA*], the upstream region of the *pitA* gene, was modified by replacement of the native regulatory region with the λPL_*tac*_ promoter *ggcggtg*-*ttgaca*-*attaatcatcggctcgtataatgtgtggaat* [hybrid of λPL (*ggcggtg*) and λP*tac* (*attaatcatcggctcgtataatgtgtggaat*) promoters. λP*tac* promoter-35 sequence and λPL promoter -35 sequence, shared by the two promoters (*ttgaca*)]. To construct the PCR fragment for λRed recombination with an excisable *cat* marker and flanking sequences homologous to the *pitA* regulatory region, we used primers 14 and 15 (see Additional file 1: Table S1 for details).

#### Construction of strain MG1655 [Δ*purR*::Cm^R^ Δ*purH*::Km^R^]

To design an *E. coli* strain of MG1655 with double deletions of the *purR* and *purH* genes, MG1655 [Δ*purR*::Cm^R^] (Table 1) was used as the recipient for P1-duction of the Δ*purH*::Km^R^ cassette from the Keio collection [33]. The presence of two chromosomal modifications, Δ*purR*::Cm^R^ and Δ*purH*::Km^R^, was confirmed by PCR with primers 16–17 and 18–19, respectively (see Additional file 1: Table S1 for details).

#### Construction of the KF37-based strain with PhoBR-independent activation of Pho regulon genes

The sequence of *phoB*^DBD^, which encodes the C-terminal DNA-binding domain of PhoB [48] [specifically, (*aa* 1–3 of PhoB)-(Glu-Phe)-(*aa* 126–229 of PhoB)], was amplified by PCR using primers 20–21 (see Additional file 1: Table S1 for details) using the MG1655 chromosome as a template. Both the PCR product and integrative plasmid pAH162-Tc^R^-2Ter (Table 1) were treated with *Sal*I and *Sma*I restriction enzymes and then ligated with T4 DNA ligase. The ligation mixture was transformed into the CC118 λ*pir*+ strain (Table 1), and the resulting pAH162-Tc^R^-2Ter-*phoB*^DBD^ plasmid, containing the promoter-less *phoB*^DBD^ gene, was inserted into the *yibH* locus of the MG1655 [Δ($\upvarphi$80-*attB*) ∆*yibH*::$\upvarphi$80-*attB*] chromosome by $\upvarphi$80-Int-mediated integration. The MG1655 [∆*yibH*::Tc^R^-*phoB*^DBD^] strain was cured of the Tc^R^ marker using a pMWts-λInt/Xis helper plasmid. A P_*lac*UV5_ promoter with the Km^R^ marker was inserted upstream of the *phoB*^DBD^ gene using λRed recombination. The integrative DNA fragment was obtained by overlap extension PCR. To achieve this goal, the Km^R^ marker was amplified from a pMW118-Km^R^ template using primers 22–23 (see Additional file 1: Table S1 for details). Promoter P_*lac*UV5_ was amplified in parallel from a pML-P_*lac*UV5_-*lacI* [39] template using primers 24–25 (see Additional file 1: Table S1 for details). Extension PCR was performed by mixing the two initially amplified DNA fragments and using primers 22 and 26 (see Additional file 1: Table S1 for details). λRed recombination was applied to the MG1655 [∆*yibH*::*phoB*^DBD^] strain, and the resulting MG1655[∆*yibH*::Km^R^-P_*lac*UV5_-*phoB*^DBD^] strain was obtained. The strains MG1655-[∆*yibH*::Tc^R^-*phoB*^DBD^] and MG1655 [∆*yibH*::Km^R^-P_*lac*UV5_-*phoB*^DBD^] were used as donors to individually transfer the constructs ∆*yibH*::Tc^R^-*phoB*^DBD^ and ∆*yibH*::Km^R^-P_*lac*UV5_-*phoB*^DBD^ into strain KF37 by P1-duction.

## Results

### AICAR controls its own formation in the His biosynthetic pathway

**Fig. 2:**
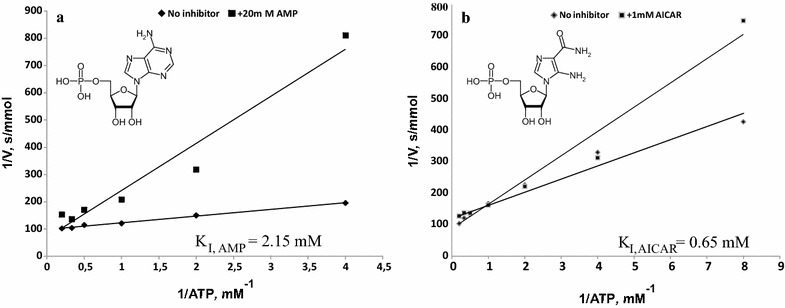
Lineweaver–Burk plots. **a** Inhibition of HT-HisG^WT^ by AMP. **b** Inhibition of HT-HisG^WT^ by AICAR. Chemical structures of AMP (**a**) and AICAR (**b**)

The enzymatic activity of HisG is subject to (i) feedback inhibition by His as the final pathway product, (ii) competitive inhibition by PR-ATP as a reaction product [6], and (iii) competitive inhibition by ADP and AMP, compounds that are structurally similar to its ATP substrate. The synthesis of each His molecule is accompanied by the release of one molecule of AICAR, an intermediate of de novo purine biosynthesis. In turn, AICAR is a well-known natural analog of AMP and can substitute for it in a number of biochemical reactions [49, 50]. Considering the structural similarity between AMP and AICAR (Fig. 2), not only AMP but also AICAR might negatively affect HisG enzymatic activity.

To test this hypothesis, we measured the HisG activity of two purified His_6_-tagged enzymes, the wild type (HT-HisG) and a Fbr-mutant variant (HT-HisG^E271K^) that is resistant to feedback inhibition by His, in the presence of either AMP or AICAR (Fig. 2, Table 2). Figure 2 shows that the partial inhibition of HisG was detected for both tested compounds, but inhibition by AICAR was significantly more pronounced and was detected at low concentrations at which inhibition by AMP was nearly absent, that is in consistence with the literature data [51] (see Table 2). The dephosphorylated form of AICAR, (AICAr), does not affect HisG activity (data not shown). His biosynthesis is thus regulated not only by histidine itself as the terminal product of this pathway but also by AICAR as an AMP analog. Moreover, the biosynthesis of AICAR per se is under negative control via the initial step of His pathway, which is catalyzed by HisG.

**Table 2:** Specific activity of purified His_6_-tagged wild-type and feedback-resistant ATP-phosphoribosyltransferases and their inhibition by AMP and AICAR

Enzyme	ATP-phosphoribosyltransferase activity, µmol/min/mg					Inhibition, %			
	–	AMP		AICAR		AMP		AICAR	
	–	1 mM	20 mM	0.5 mM	1 mM	1 mM	20 mM	0.5 mM	1 mM
HT-HisG	184 ± 9	195 ± 1	120 ± 15	151 ± 9	120 ± 5	0	35	18	35
HT-HisG^E271K^	120 ± 1	129 ± 12	nm	67 ± 4	58 ± 8	0	nm	44	52

Average data of 3 independent experiments are represented

*nm* not measured

AMP inhibits HisG activity due to competition with one of HisG’s native substrates, ATP [41]; AICAR could therefore be similar to AMP and thus exhibit an even stronger competitive inhibition of HisG.

### Enhancement of AICAR conversion to ATP to intentionally overproduce His

The formation of AICAR by His biosynthetic enzymes is negatively controlled by AICAR inhibition of HisG. His production is thus directly regulated by this intermediate of purine biosynthesis, and a reduction in the AICAR pool due to its recycling to ATP may be one important way to overcome the bottleneck in His overproduction (Fig. 1). At a minimum, overexpressing *purH* and *purA* genes is necessary to enhance AICAR conversion to ATP (see Fig. 1). For *purH* overexpression, its native regulatory region in the chromosome of the MG1655 wild-type strain was replaced with the “strong” constitutive λP_L_ promoter via λRed mediated recombination, and the MG1655 [Cm^R^-P_L_-*purH*] strain was obtained (see Methods for details).

To increase *purA* gene expression, we specifically introduced an additional copy of the corresponding gene into the IS 5.11 locus of the MG1655 chromosome (as described in “Methods” section), followed by P1-duction of the Cm^R^-P_*tac*21_-*purA* cassette into several strains of interest. One of the obtained Cm^R^-ductants, an MG1655-derivative that had earlier passed through several stages of selection for increased His overproduction, was used in the present study as the donor for P1-duction of the *purA*-carrier marked cassette. This cassette was P1-duced into the His-overproducing strain KF37 [13], followed by evaluation of His accumulation by the obtained Cm^R^-ductants in test tube fermentations. Two groups of P1-ductants were selected according to their ability to overproduce His. The members of the first (main) group (more than 70% of the tested P1-ductants) increased His accumulation by approximately 20% over its levels in the initial KF37 strain. The members of the second (minor) group also exceeded KF37 in His overproduction, but the difference was approximately 6% (Table 3). Direct sequencing of the chromosomal regions flanking the bacterial locus IS5.11, the point of integration of the *purA*-carrier cassette in the P1-ductants obtained on the basis of KF37 and in the strain used as the donor for P1-duction, confirmed that the difference in His accumulation did not correspond to the structure of the cassettes, which were the same, in full agreement with the proposed design. The difference could be explained by the nonsense mutation that inactivated the *pitA* gene being closely coupled with the point of *purA* integration in the initial donor and in the tested P1-ductants that produced more His. This mutation was absent in the tested chromosomes of the P1-ductants from the second group. Moreover, the absence or presence of the mutation leading to *pitA* inactivation was confirmed by allele-specific PCR for the different members of the second and the first groups of P1-ductants, respectively (see Additional file 1: Table S1 for details, primers 1–3). One of the variants from the first group of P1-ductants that produced more His was cured of the Cm^R^-marker due to Xis/Int-mediated recombination; the obtained markerless strain was assigned as EA79 and used for the following improvement process.

**Table 3:** Effect of PitA inactivation on His production by the strain KF37 [IS5.11::CmR-P_tac21_-*purA pitA*^−^]

Strain	*pitA* allele	OD_540_	His, g/L	His, %
KF37	*pitA* ^+^	14.9 ± 0.4	3.3 ± 0.1	100
KF37 [IS5.11::Cm^R^-P_*tac*21_-*purA, pitA*^+^]	*pitA* ^+^	16.1 ± 0.1	3.5 ± 0.1	106
KF37 [IS5.11::Cm^R^-P_*tac*21_-*purA pitA*^−^]	*pitA* ^−^	15.6 ± 0.4	4.0 ± 0.1	121

Average data from 8 independent experiments are represented

**Fig. 3:**
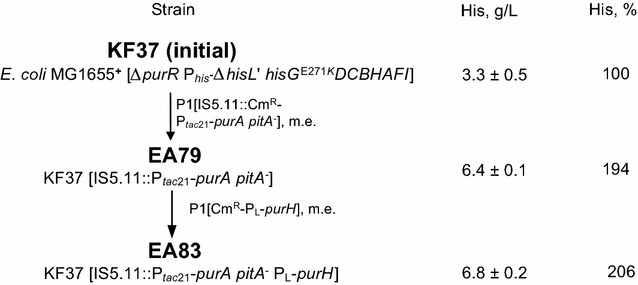
Genealogy of His-producing strains with enhanced AICAR conversion into ATP. *me* marker elimination. Average data from 10 independent experiments are represented

Figure 3 shows that the overexpressed *purH*-carrier cassette was P1-duced from the strain MG1655 [Cm^R^-P_L_-*purH*] into EA79 using Cm^R^ as a selective marker, and the marker was then excised to yield the EA83 strain, which accumulated 10% more His than EA79. In full accordance with the earlier supposition, the possible decrease in the intracellular AICAR pool due to the overexpression of *purA* and *purH* genes thus actually increased the level of His accumulation. At the same time, the occasional co-transduction of the mutation-inactivated *pitA* gene closely located to a P1-duced cassette had a much greater positive effect on His production than the overexpression of *purA* itself (see Table 3).

### PitA deficiency positively affects l-His production

To analyze the individual effect of PitA transporter elimination on His production, we deleted the corresponding gene in strain KF37, and the positive influence of PitA deficiency was confirmed (Table 4). In opposite, enhancement PitA transporter by introduction of the Cm^R^-P_L*tac*_-*pitA* expression cassette, harboring overexpressed *pitA* gene under the strong λP_L*tac*_ promoter control, into the His-producing strain KF37 lead to the drastically reduction of growth rate and His accumulation. The inactivation of *pitB,* which encodes a minor metal phosphate/H^+^ symporter, alone or in addition to the elimination of PitA did not influence the level of His accumulation (data not shown).

**Table 4:** Influence of PitA deficiency and enhancement on His production

Strain	OD_540_	His, g/L	His accumulation, %
KF37	14.9 ± 0.3	3.4 ± 0.1	100
KF37 [Δ*pitA*::Km^R^]	11.3 ± 0.5	3.8 ± 0.3	112
KF37 [Cm^R^-P_L*tac*_-*pitA*]	7.1 ± 0.2	1.4 ± 0.2	41

Average data from 6 independent experiments are represented

**Fig. 4:**
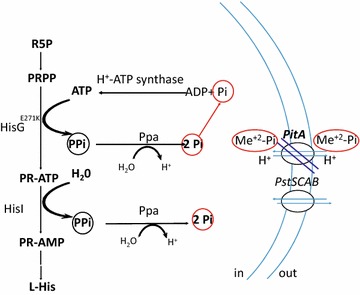
Schematic view of the putative role of PitA deficiency in the cellular P_i_/PP_i_ balance and histidine biosynthetic pathway. *ATP* adenosine triphosphate, *PP*_*i*_ inorganic pyrophosphate, *P*_*i*_ inorganic orthophosphate, *Me*^*2*+^*-P*_*i*_ metal phosphate complexes, *PRPP* phosphoribosyl pyrophosphate, *PR-ATP* phosphoribosyl-ATP, *PR-AMP* phosphoribosyl-adenosine monophosphate, *ADP* adenosine diphosphate, *L-His* l-histidine, *HisG*^*E271K*^ feedback-resistant ATP-phosphoribosyltransferase, *HisI* phosphoribosyl-AMP cyclohydrolase/phosphoribosyl-ATP pyrophosphatase, *Ppa* inorganic pyrophosphatase, *PitA* low affinity P_i_ transport system, *PstSCAB* phosphate specific transport system

The nature of this phenomenon is unclear, although several hypotheses concerning the positive influence of Δ*pitA* on His production were generated on the basis of the known properties of the PitA Me^2+^-P_i_ transporter. We supposed that the deletion of *pitA* can decrease import of P_i_ and Zn^2+^. The decreased import of Zn^2+^ may increase availability of Mg^2+^ for the efficient PPase-mediated PP_i_ hydrolysis [24, 52] and result in the promotion of PP_i_-generating reactions, including those of the His biosynthetic pathway (Fig. 4, see “Discussion” for details). In turn, the His biosynthetic proteins HisG and HisI are PP_i_-releasing enzymes, and a metabolic significance of their function could thus be coupling with PPase activity to increase the intracellular P_i_ pool.

### Influence of Δ*pitA* on Pho regulon induction and P_i_ uptake

To test the supposition concerning the decrease in inorganic phosphate import and probable alterations of the P_i_ pool in the absence of PitA, we evaluated the possible influence of PitA inactivation on Pho regulon gene activation. The activity of the bacterial alkaline phosphatase (EC 3.1.3.1, PhoA, the product of the *phoA* gene that belongs to the *E. coli* Pho regulon [53–57]) was measured for this purpose. Growth in the MOPS-based minimal medium and P_i_ consumption were initially investigated for the wild-type strain MG1655, the His-producing strain KF37 and their respective *pitA*-deficient variants (see Additional file 3: Figure S2A and B). PhoA enzymatic activity was analyzed 24 h after P_i_ exhaustion. PitA transporter inactivation did not significantly change the growth efficiency and capability of P_i_ uptake of either MG1655 or KF37 under the tested conditions (see Additional file 3: Figure S2A), indicating that the total P_i_ uptake could be only slightly dependent on activity of PitA transporter. The insignificant effect of PitA transporter inactivation on capability of P_i_ uptake confirmed the knowledge concerning the main formation of intracellular P_i_ pools in *E. coli*. The variety of recent data show that the Pst system, but not PitA system, serves as the primary P_i_ transporter when P_i_ is in excess [22].

**Fig. 5:**
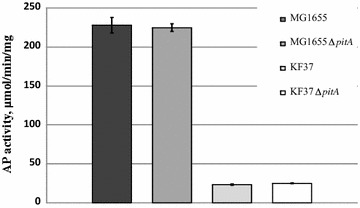
AP enzymatic activity under conditions of P_i_ limitation in His-producing and non-producing *E. coli* strains with different *pitA* alleles. MG1655, wild-type MG1655; MG1655 ∆*pitA*, MG1655 [Δ*pitA*::Km^R^]; KF37, MG1655^+^ [Δ*purR* P_*his*_-Δ*hisL′hisG*^E271K^*DCBHAFI*]; KF37 *∆pitA*, KF37 [Δ*pitA*:: Km^R^]. Average data from 3 independent experiments are represented, and error bars show the standard deviation (SD). PhoA enzymatic activity was analyzed 24 h after P_i_ exhaustion

**Fig. 6:**
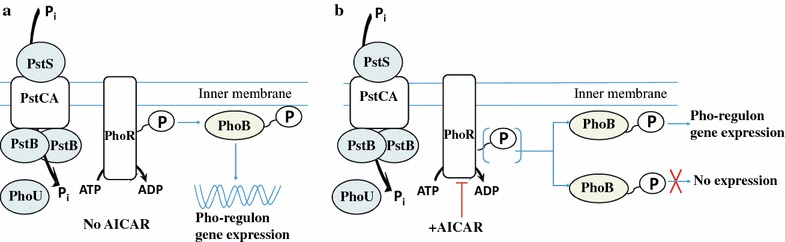
Proposed model of Pho regulon expression control. **a** Activation of the Pho regulon during P_i_ limitation. **b** Partial deactivation of the Pho regulon during P_i_ limitation in the presence of AICAR. *PhoR* histidine kinase, *PhoB* response regulator, *PstSCAB* phosphate-specific ABC transporter, *PhoU* negative regulator of PhoR, *AICAR* 5-aminoimidazole-4-carboxamide ribonucleotide, *P*_*i*_ inorganic phosphate

Interestingly, PhoA activity after P_i_ depletion was practically absent in the His-producing strain KF37, regardless of the *pitA* allele status and in contrast to the wild-type strain (Fig. 5). In the framework of the existing model of the control of Pho regulon [22, 58, 59], the hypothesis of possible AICAR negative influence on Pho regulon, in particular, PhoR kinase activity, was proposed. As known, *E. coli* PhoR histidine kinase has catalytic ATP-binding CA domain [58]. Pho regulon repression in case of His-producing strains could be explained by a reduction of PhoR’s ability to ATP-dependently autophosphorylate, which is necessary for the subsequent manifestation of its kinase activity in relation to PhoB when the Pho regulon activates (see Fig. 6, adapted from Hsieh and Wanner [22]). This reduction is probably based on an inhibition of ATP-dependent PhoR by AMP structural analog, AICAR, whose pool might be increased in His-producing strains.

### Changes in the activity of PhoA in response to AICAR oversynthesis

To study whether the Pho regulon is repressed by AICAR, we constructed a strain with elevated intracellular levels of this metabolite and tested the activation of the Pho regulon in this strain under P_i_-limiting conditions. Overproduction of AICAR was stimulated by inactivation of the PurR, a repressor of purine biosynthetic genes, and AICAR metabolism was simultaneously prevented by *purH* gene deletion. The AICAR pool was increased in the resulting double mutant strain MG1655 [Δ*purR*::Cm^R^ Δ*purH*::Km^R^], and this increase was detected by the appearance of AICAr, the dephosphorylated form of AICAR, in the culture broth (Table 5).

**Table 5:** Alkaline phosphatase (PhoA) activity under conditions of AICAR overproduction

Strain	AICAr, mg/L	PhoA activity, µmol/min/mg
MG1655 (wild-type)	< 0.1	410 ± 7
MG1655Δ*purR*::Cm^R^ Δ*purH*::Km^R^	7.6	261 ± 1

PhoA enzymatic activity was analyzed 2 h after P_i_ exhaustion. Average data of PhoA activity from 3 independent experiments are represented

We cultivated the *E. coli* wild-type strain and the strain overproducing AICAR under P_i_-limiting conditions (their growth and P_i_ exhaustion kinetics are shown in Additional file 4: Figure S3). PhoA enzymatic activity was analyzed 2 h after P_i_ exhaustion. As expected, the level of PhoA alkaline phosphatase activity was appreciably less in the AICAR-overproducing strain than the wild-type strain (Table 5) but significantly higher than the KF37-based His-producing strains (compared in Fig. 5). These results collectively supported the hypothesis that an increased AICAR pool (in AICAR- and His-overproducing strains) was the main reason for the decreased expression of the Pho regulon genes and were likely due to AICAR inhibiting PhoR autophosphorylation, which in turn restricted the phosphorylation of PhoB.

### P_i_-independent activation of *phoA* gene expression in the His-producing strain KF37

**Fig. 7:**
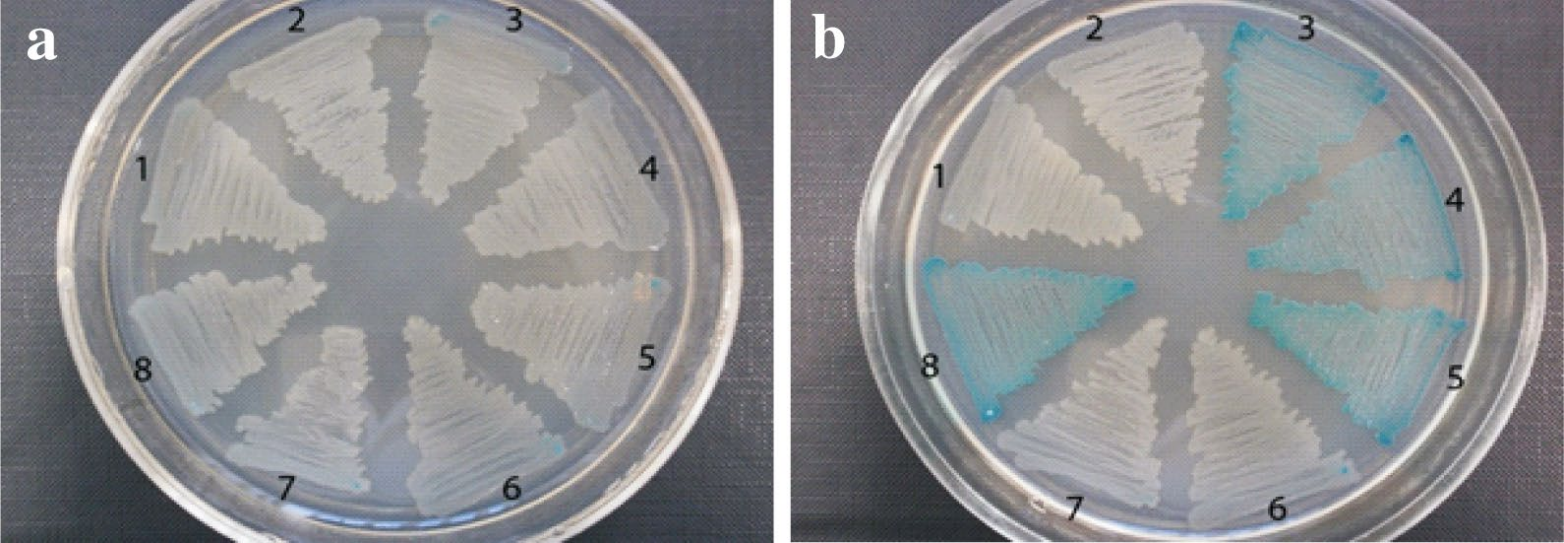
Strains expressing the *phoB*^DBD^ gene. **1** KF37 [Δ*yibH*::Tc^R^-*phoB*^DBD^], **2** KF37 [∆*yibH*::Tc^R^-*phoB*^DBD^], **3** KF37 [Δ*yibH*::Km^R^-P_*lac*UV5_-*phoB*^DBD^]. **4** KF37 [Δ*yibH*::P_*lac*UV5_-*phoB*^DBD^], **5** KF37 [Δ*yibH*::P_*lac*UV5_-*phoB*^DBD^], **6** KF37, **7** MG1655 and **8** MG1655 [Δ*yibH*::P_*lac*UV5_-*phoB*^DBD^] after overnight incubation on LB agar supplemented with 50 mg/L BCIP without (**a**) and with (**b**) 500 µM IPTG addition

The expression of a truncated PhoB regulator containing only the DNA-binding C-terminal domain (PhoB^DBD^ [48, 57, 60]) was examined to confirm the normal functionality of the *phoA* gene in the KF37 His-producing strain. The expression of PhoB^DBD^ provides a P_i_-independent activation of the Pho regulon [48]. PhoA synthesis in the growing cells was easily visualized as blue-colored colonies on plates containing medium supplemented with the specific chromogenic substrate 5-bromo-4-chloro-3-indolyl phosphate (BCIP). Derivatives of the His-producing strain KF37 that also harbored the cassette containing the PhoB^DBD^ gene under the control of the IPTG-inducible promoter P_*lac*UV5_ synthesized PhoA after induction, as did derivatives of the wild-type strain MG1655 harboring the same cassette (Fig. 7). This finding supports the hypothesis that His overproduction affects the function of the two-component signal transduction system PhoBR rather than the Pho regulon genes themselves.

## Discussion

AICAR is formed during His biosynthesis and in the purine biosynthetic pathway [14, 29]. The negative effects of intracellular AICAR accumulation on different aspects of bacterial metabolism, such as adenosine homeostasis, gluconeogenesis, and thiamine synthesis, were investigated in *S. enterica* [61]. Moreover, several studies suggested that AICAR accumulation represses not only purine biosynthesis [62] but also one or more steps of the His pathway in *S. cerevisiae* [63]; however, the mechanism underlying such effects remains unclear. On the other hand, AICAR acts as a natural analog of AMP and can substitute for it in some enzymatic reactions. In turn, AMP inhibits enzymes involved in de novo purine and His biosynthetic pathways in which AICAR is formed as an intermediate [41]. We therefore hypothesized that AICAR controls its own formation, particularly in the His biosynthetic pathway. This supposition was investigated in this study by an examination of the possible influence of AICAR on the conversion of PRPP and ATP to PR-ATP, which is accomplished by HisG. The inhibitory effect of AICAR on HisG activity in vitro was demonstrated for the first time. The inhibition of HisG by high concentrations of AMP was also confirmed. Both tested substances compete with ATP in HisG-catalyzed reactions, suggesting that similar mechanisms are present for enzyme-ligand interactions. Figure 2 shows that AICAR was an even more effective inhibitor than AMP. AICAR thus controls its own formation with regard to the His biosynthetic pathway. Similar negative control may occur during AICAR formation via de novo purine biosynthesis; in this case, AICAR might control PurF enzyme activity, which is known to be negatively affected by AMP. In addition, some other possibilities for His biosynthesis regulation with participation of AICAR could be investigated. It is known that AICAR is identified as an alarmone that senses e.g. 10-formyl-tetrahydroflate deficiency in bacteria and activates a conserved riboswitch to regulate the expression of tetrahydrofolate genes involved in one-carbon metabolism [64, 65]. Therefore, it would be particularly interesting to study further the other aspects of the influence of AICAR on His biosynthesis, especially, considering close relationship of this biosynthetic pathway with one-carbon metabolism.

His overproduction cannot be engineered solely by enabling the resistance of the first biosynthetic enzyme, HisG, to feedback inhibition (see e.g., [13]). The effective metabolism of AICAR to ATP is also required to prevent the undesirable accumulation of AICAR, which is a HisG inhibitor, and to enhance the supply of ATP, which is an essential component for the synthesis of this amino acid. The effectiveness of such an approach was tested, yielding the *E. coli* His-overproducing strain EA83, which enhances AICAR regeneration into ATP (Fig. 3).

Furthermore, we found that a deficiency in PitA of the Me^2+^-P_i_ transport system considerably increased His accumulation (Table 3). A spontaneous mutation that inactivated the *pitA* gene was accidentally selected during the process of constructing the His-overproducing strains. The mechanism underlying this effect is not clear. Nevertheless, effective Zn^2+^-P_i_ import via the PitA transporter under conditions of excess extracellular phosphate and Zn^2+^ ions may restrict the total PP_i_ hydrolysis efficacy, thereby inhibiting the His biosynthetic pathway reactions (Fig. 4). In *E. coli*, the hydrolysis of pyrophosphate (PP_i_) to P_i_ is catalyzes by inorganic pyrophosphatase (PPase, EC 3.6.1.1, the *ppa* gene product) in the presence of different divalent metal ions (e.g., Mg^2+^, Zn^2+^, Mn^2+^, and Co^2+^), but only using Mg^2+^ as physiological activator does PPase significantly increase the efficiency and specificity of PP_i_ hydrolysis [52, 66, 67]. However, this specificity is lost when transition metal ions such as Zn^2+^, Mn^2+^ and Co^2+^ are used as cofactors, leading to markedly increased activity against polyphosphates [66] and the ability to hydrolyze organic tri- and diphosphates such as ATP and ADP [68, 69]. To test this hypothesis, we examine the effect of PitA deficiency on capability of Pi. Deletion of the *pitA* gene did not significantly decrease the import of P_i_ in the mutant strains from its levels in the PitA^+^ *E. coli* strains (P_i_ consumption values for both types of strains were within experimental uncertainty, see Additional file 3: Figure S2). These results correlate well with main formation of intracellular P_i_ pools by a basal level of Pst transporter activity in conditions of excess extracellular P_i_ [22]. The intracellular Pi pool was thus expected to be only slightly lower in *pitA* mutants than wild type. However, the deletion of *pitA* could additionally decrease import of Zn^2+^ ions and perhaps significantly increase the portion of PPase in complex with Mg^2+^, which improves enzyme–substrate specificity [70]. Decreasing the import of both P_i_ and Zn^2+^ could thus promote PP_i_-releasing reactions (in the His biosynthetic pathway, in particular) due to subsequent thermodynamically defensible hydrolysis of PP_i_ to P_i_.

To elucidate the roles of the other known Zn^2+^ transport systems during His production, we tested variants of a His-producing strain with a deleted *znuA* gene, which encodes the substrate-binding component of the ZnuABC high-affinity zinc uptake system [71], for His accumulation; the *znuA* deletion did not affect cell growth and had no positive effect on the rate of His accumulation (data not shown). These findings support the supposition that the effect of PitA deficiency on His production could not be explained solely by a decrease in Zn^2+^ uptake.

The last new feature that was detected for the His-producing strains in the present study was the significantly decreased Pho regulon activation in the growing cells after the depletion of the P_i_ that had been initially added in the cultural medium. This effect may have been based on the two-component PhoBR system and was likely to depend on AICAR-dependent inhibition of the PhoR autophosphorylation process. Indeed, the level of PhoA activation in the specially constructed AICAR-overproducing strain was lower than that of the wild-type strain, but this effect was not so pronounced as in His-producing cells.

## Conclusions

Both the native HisG and feedback resistant HisG^E271K^ from *E. coli* were shown to be sensitive to inhibition by AICAR. Accordingly, the production of l-histidine was improved in *E. coli* through the construction of strain EA83. In this strain, the AICAR regeneration pathway was enhanced via *purA* and *purH* gene overexpression and PitA-dependent phosphate/metal transport system was inactivated. Moreover, PhoA enzymatic activity was shown to be almost absent in the His-producing strain after P_i_ depletion in the medium, leading to the hypothesis that Pho regulon depression is associated with the inhibitory effect of AICAR, which is produced during the excessive production of His and acts as a regulatory molecule.

## Additional files

**Additional file 1: Table S1.** PCR primers used in this study.

**Additional file 2: Figure S1.** Expression and purification of HT-HisG^WT^ (wild-type) and mutant HT-HisG^E271K^. (**a**) SDS-PAGE of total protein containing HT-HisG^WT^ and HT-HisG^E271K^ before purification. M, protein molecular weight standards; HisG^WT^, crude cell lysate of BL21(DE3)/pET15-hisG^WT^ after IPTG induction; HisG^E271K^, crude cell lysate of BL21(DE3)/pET15-hisG^E271K^ after induction. (**b**) SDS-PAGE of the two purified HT-HisG^WT^ proteins. M, protein molecular weight standards; HisG^WT^, HT-HisG^WT^ after purification; HisG^E271K^, HT-HisG^E271K^ after purification.

**Additional file 3: Figure S2.** Effect of PitA deficiency on growth (**a**) and P_i_ uptake (**b**) during P_i_ starvation. (**a**) Growth of wild-type MG1655; MG1655 ∆*pitA*, MG1655 [Δ*pitA*::Km^R^], KF37; KF37 ∆*pitA*, KF37 [Δ*pitA*::Km^R^] strains. Growth of the KF37 His-producing strains was better during the initial period in MOPS medium, but the final optical density was the same as or lower than that of wild-type. The better initial growth of the KF37 strain can be explained by its effective sugar consumption, which was also monitored (data not shown) and was found to be exhausted at 17 h for the His-producing strain compared to 22 h for the wild-type strain (data not shown). These results confirm the measured kinetics of P_i_ uptake (**b**) from the medium; as expected, the rate of P_i_ uptake was higher for the His-producing strains under such conditions. Error bars show the standard deviation (SD).

**Additional file 4: Figure S3.** Effect of AICAR on growth (**a**) and P_i_ uptake (**b**) during P_i_ starvation of wild-type MG1655; MG1655 *∆purR ∆purH*, MG1655 [∆*purR*::Cm^R^ ∆*purH*:: Km^R^]. The vertical arrow indicates the sampling time for the measurement of AP enzymatic activity. Error bars show the standard deviation (SD).

## Abbreviations

His: l-histidine
Fbr: feedback resistance
PRPP: phosphoribosyl pyrophosphate
AS: adenylosuccinate
AICAR: 5-aminoimidazole-4-carboxamide ribonucleotide, phosphorylated form, ZMP
AICAr: 5-aminoimidazole-4-carboxamide-1-beta-d-ribofuranosyl-5′-monophosphate, dephosphorylated form
FAICAR: 5′-phosphoribosyl-5-formamido-4-imidazole carboxamide
P_i_: inorganic phosphate
PP_i_: inorganic pyrophosphate
Pst: PstSCAB, phosphate specific transport
Pit: phosphate inorganic transport
Me^2+^-P_i_: metal phosphate (MeHPO_4_) complexes
TLC: thin layer chromatography
HT-HisG: His_6_-Tag-HisG
Da: Dalton
OD: optical density
SD: standard deviation
PCR: polymerase chain reaction
OE-PCR: overlap extension PCR
SDS-PAGE: sodium dodecyl sulfate polyacrylamide gel electrophoresis
pNPP: *p*-nitrophenyl-phosphate
AP: alkaline phosphatase
Pho regulon: phosphate regulon
IPTG: isopropyl β-d-1-thiogalactopyranoside
BCIP: 5-bromo-4-chloro-3-indolyl phosphate disodium salt
