# Supplementary material for: Specific features of l-histidine production by Escherichia coli concerned with feedback control of AICAR formation and inorganic phosphate/metal transport
Source: Microb Cell Fact. 2018 Mar 15;17:42. doi: 10.1186/s12934-018-0890-2 (PMC5852967; doi:10.1186/s12934-018-0890-2)
Supplement: Supplementary file 1 — Additional file 1: Table S1. PCR primers used in this study. [file 12934_2018_890_MOESM1_ESM.docx]

| No | Sequence (5’→3’) | Description |
| --- | --- | --- |
| 1 | gattatctggaacctgggtaccca | Testing for the specific mutant allele presence into the chromosome, allele-specific PCR |
| 2 | gattatctggaacctgggtacccg | Testing for the specific mutant allele presence into the chromosome, allele-specific PCR |
| 3 | cgacgttgttgatggcatca | Testing for the specific mutant allele presence into the chromosome, allele-specific PCR |
| 4 | catatgacagacaacactcgttta | Amplifying the fragment N-*Nde*I*-hisG* with N-NdeI restricton site for cloning to pET15b plasmid |
| 5 | ggatcctcactccatcatcttctcaat | Amplifying the fragment N-*Nde*I*-hisG* with N-*Nde*I restricton site for cloning to pET15b plasmid |
| 6 | gaaggagttttgaaaaatgggtaacaacgtcgtcgt | Amplifying the fragment with the native SD followed by the additional sequence *gaaggag* for PurA cloning to pMW119-P*_lac_*-*lacI* plasmid |
| 7 | ttacgcgtcgaacgggtcgc | Amplifying the fragment with the native SD followed by the additional sequence *gaaggag* for PurA cloning to pMW119-P*_lac_*-*lacI* plasmid |
| 8 | acgacgacgttgttacccatttttcaaaactccttccgctcacaattccacacattatacgagc | Amplifying the fragment λ-*attB*::Cm^R^ with homologous regions for λP*_tac_*_21_ promoter integration upstream *purA* locus |
| 9 | ggatattatcgtgaggatgcgtcatcgccattaattcgctcaagttagtataaaaaagctgaac | Amplifying the fragment λ-*attB*::Cm^R^ with homologous regions for λP*_tac_*_21_ promoter integration upstream *purA* locus |
| 10 | gagcagagcgcggcggactggacgacgttgttgcatagttagttctccttccggccaatgcttcg | Amplifying the fragment λ-*attB*::Cm^R^ with homologous regions for λP_L_ promoter integration upstream *purH* locus |
| 11 | gtagacagcagctccacacc | Amplifying the fragment λ-*attB*::Cm^R^ with homologous regions for λP_L_ promoter integration upstream *purH* locus |
| 12 | cgccgcgttcatgtcctcaaaatggcgtaacgtccttgaagcctgcttttttatactaagttgg | Amplifying the fragment λ-*attB*::Km^R^ with homologous regions for integration in *pitA* locus |
| 13 | gtacgattacaggaactgcaaggagagccagtacagcgctcaagttagtataaa aaagctgaac | Amplifying the fragment λ-*attB*::Km^R^ with homologous regions for integration in *pitA* locus |
| 14 | aaatgtagcataggacgttacgccattttgaggacacgctcacaattccacacat | Amplifying the fragment λ-*attB*::Cm^R^ with homologous regions for λP_L_*_tac_* hybrid promoter integration upstream *pitA* locus |
| 15 | tcagtggtcgcaccagcatcaatattactaaaaggacgctcaagttagtataaaaaagct | Amplifying the fragment λ-*attB*::Cm^R^ with homologous regions for λP_L_*_tac_* hybrid promoter integration upstream *pitA* locus |
| 16 | ttaaggtcggtgctcatcaag | Testing for the *purR* locus deletion into the chromosome |
| 17 | caacagtactgcgatgagtg | Testing for the *purR* locus deletion into the chromosome |
| 18 | aggcgcattatagggagttc | Testing for the *purH* locus deletion into the chromosome |
| 19 | attccggggatccgtcgacc | Testing for the *purH* locus deletion into the chromosome |
| 20 | atcattgtcgacatggcgagagaattcatggcggtggaagaggtgat | Amplifying the fragment contained *phoB*^DBD^ with *Sal*I restricton site for cloning to pAH162-*λattL*-*tetA*-*tetR*-*λattR*-2Ter |
| 21 | cttgaacccgggttaaaagcgggttgaaaaacgatatc | Amplifying the fragment contained *phoB^DBD^* with *Sma*I restricton site for cloning to pAH162-*λattL*-*tetA*-*tetR*-*λattR*-2Ter |
| 22 | ctttttgcgtggccagtgccaagcttgcatgcctgctgaagcctgcttttttatactaagttgg | Amplifying the excisable marker Km^R^ for OE-PCR with P*_lac_*_UV5_ promoter |
| 23 | ctcactgcccgcagatctcgctcaagttagtataaaaaagctgaac | Amplifying the excisable marker Km^R^ for OE-PCR with P*_lac_*_UV5_ promoter |
| 24 | cttttttatactaacttgagcgagatctgcgggcagtgagcgc | Amplifying P*_lac_*_UV5_ promoter for OE-PCR with the excisable marker Km^R^ |
| 25 | atgtatatctccttcttaaagttaaacaaaattatttctagatcctgtgtgaaattgttatccgc | Amplifying P*_lac_*_UV5_ promoter for OE-PCR with the excisable marker Km^R^ |
| 26 | aatcacctcttccaccgccatgaattctctcgccatatgtatatctccttcttaaagttaaacaaaattatt | OE-PCR Amplifying Km^R^-P*_lac_*_UV5_ promoter fuse |
