# Supplementary figures and images for: Specific features of l-histidine production by Escherichia coli concerned with feedback control of AICAR formation and inorganic phosphate/metal transport
Source: Microb Cell Fact. 2018 Mar 15;17:42. doi: 10.1186/s12934-018-0890-2 (PMC5852967; doi:10.1186/s12934-018-0890-2)

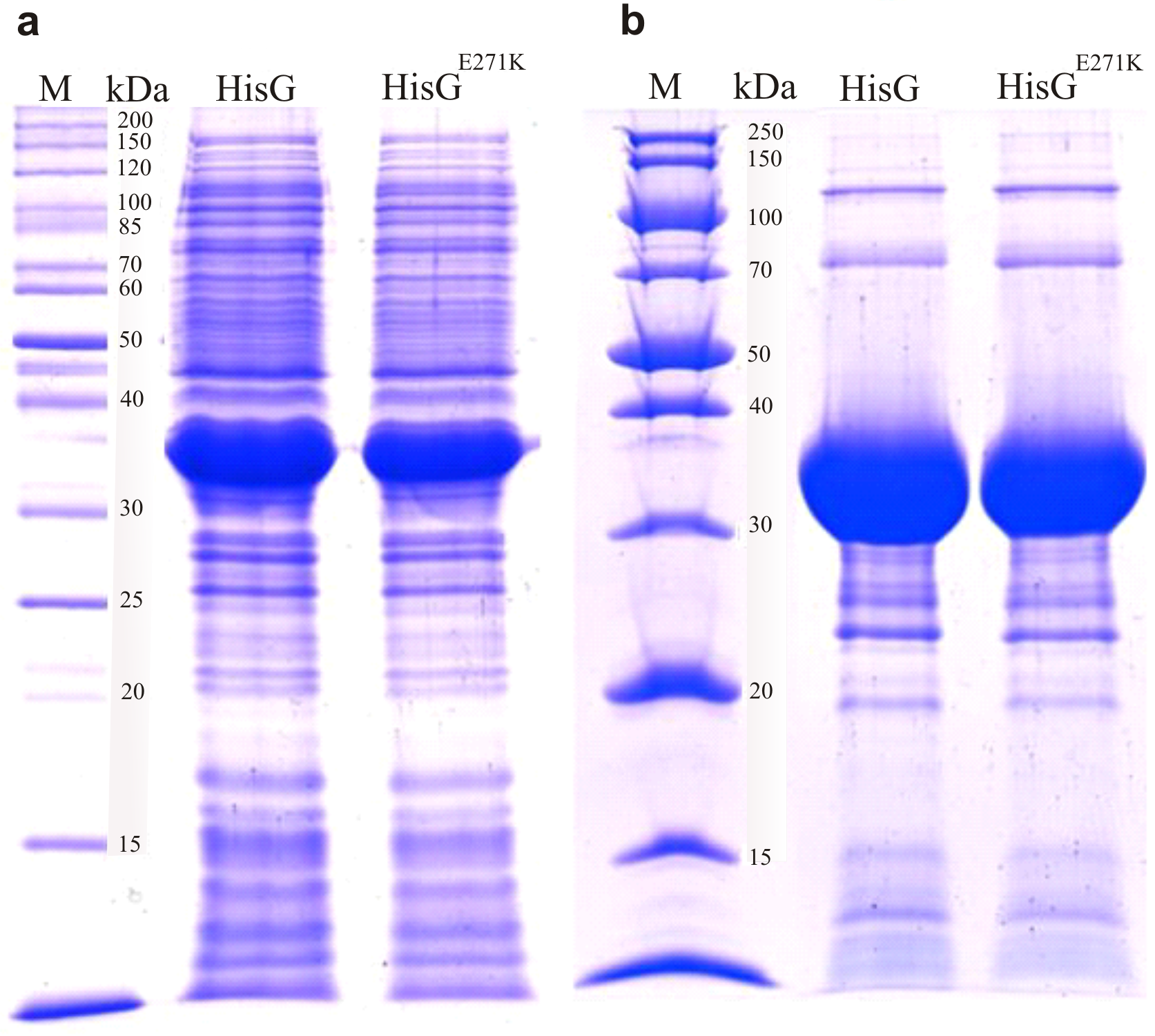

Supplement: Supplementary file 2 — Additional file 2: Figure S1. Expression and purification of HT-HisGWT (wild-type) and mutant HT-HisGE271K. (a) SDS-PAGE of total protein containing HT-HisGWT and HT-HisGE271K before purification. M, protein molecular weight standards; HisGWT, crude cell lysate of BL21(DE3)/pET15-hisGWT after IPTG induction; HisGE271K, crude cell lysate of BL21(DE3)/pET15-hisGE271K after induction. (b) SDS-PAGE of the two purified HT-HisGWT proteins. M, protein molecular weight standards; HisGWT, HT-HisGWT after purification; HisGE271K, HT-HisGE271K after purification. [file 12934_2018_890_MOESM2_ESM.tif]

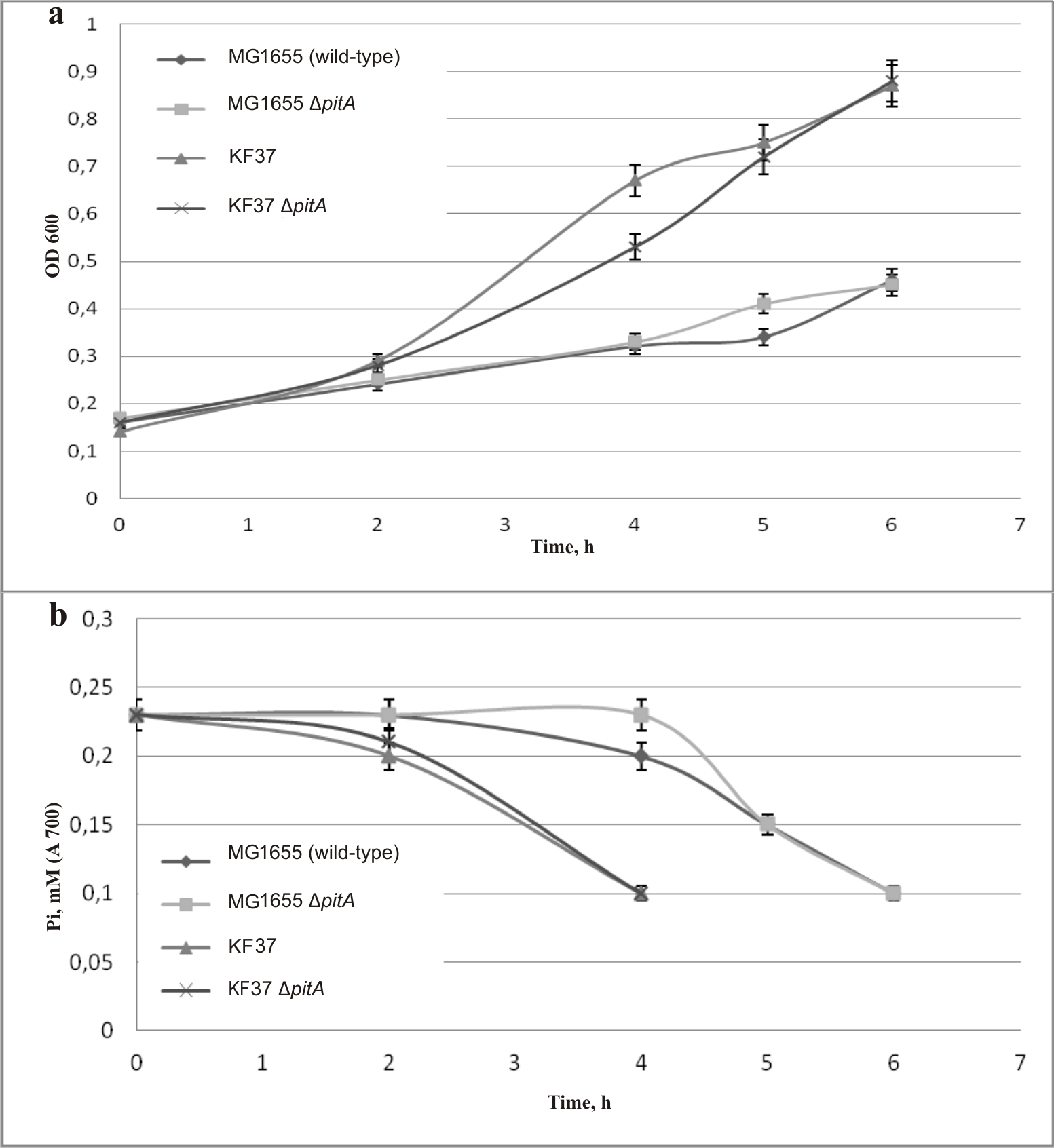

Supplement: Supplementary file 3 — Additional file 3: Figure S2. Effect of PitA deficiency on growth (a) and Pi uptake (b) during Pi starvation. (a) Growth of wild-type MG1655; MG1655 ∆pitA, MG1655 [ΔpitA::KmR], KF37; KF37 ∆pitA, KF37 [ΔpitA::KmR] strains. Growth of the KF37 His-producing strains was better during the initial period in MOPS medium, but the final optical density was the same as or lower than that of wild-type. The better initial growth of the KF37 strain can be explained by its effective sugar consumption, which was also monitored (data not shown) and was found to be exhausted at 17 h for the His-producing strain compared to 22 h for the wild-type strain (data not shown). These results confirm the measured kinetics of Pi uptake (b) from the medium; as expected, the rate of Pi uptake was higher for the His-producing strains under such conditions. Error bars show the standard deviation (SD). [file 12934_2018_890_MOESM3_ESM.tif]

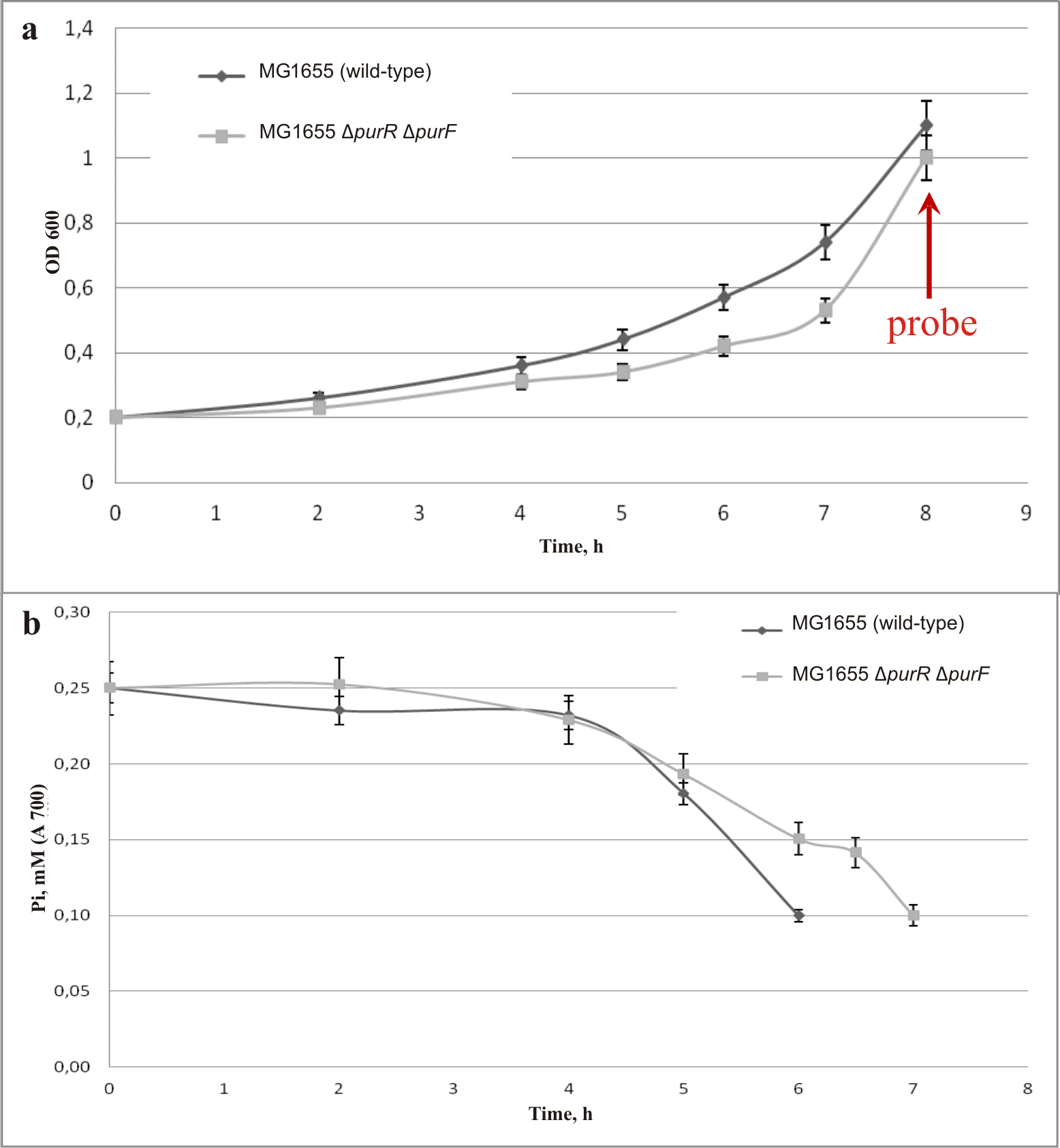

Supplement: Supplementary file 4 — Additional file 4: Figure S3. Effect of AICAR on growth (a) and Pi uptake (b) during Pi starvation of wild-type MG1655; MG1655 ∆purR ∆purH, MG1655 [∆purR::CmR ∆purH:: KmR]. The vertical arrow indicates the sampling time for the measurement of AP enzymatic activity. Error bars show the standard deviation (SD). [file 12934_2018_890_MOESM4_ESM.tif]
